# Procedural Data Processing for Single-Molecule Identification by Nanopore Sensors

**DOI:** 10.3390/bios12121152

**Published:** 2022-12-09

**Authors:** Yupeng Wang, Jianxuan Yuan, Haofeng Deng, Ziang Zhang, Qianli D. Y. Ma, Lingzhi Wu, Lixing Weng

**Affiliations:** 1 School of Materials Science & Engineering, Nanjing University of Posts and Telecommunications, Nanjing 210023, China; 2 School of Geography and Biological Information, Nanjing University of Posts and Telecommunications, Nanjing 210023, China

**Keywords:** current pulse, signal identification, nanopore, single molecular event

## Abstract

Nanopores are promising single-molecule sensing devices that have been successfully used for DNA sequencing, protein identification, as well as virus/particles detection. It is important to understand and characterize the current pulses collected by nanopore sensors, which imply the associated information of the analytes, including the size, structure, and surface charge. Therefore, a signal processing program, based on the MATLAB platform, was designed to characterize the ionic current signals of nanopore measurements. In a movable data window, the selected current segment was analyzed by the adaptive thresholds and corrected by multi-functions to reduce the noise obstruction of pulse signals. Accordingly, a set of single molecular events was identified, and the abundant information of current signals with the dwell time, amplitude, and current pulse area was exported for quantitative analysis. The program contributes to the efficient and fast processing of nanopore signals with a high signal-to-noise ratio, which promotes the development of the nanopore sensing devices in various fields of diagnosis systems and precision medicine.

## 1. Introduction

Nanopore sensing is a promising single molecular technology with the advantages of no amplification, label-free, high sensitivity, and high throughput. It has been developed in various fields such as gene sequencing, protein profiling, nanoparticle characterization, and biological particle detection [1,2,3,4,5,6]. Generally, a nanopore sensor is based on the resistive-pulse model in the electrolyte solution, where the nanoscale pore, which is drilled into a thin insulating film, is the only path of the ionic current flowing under the biased voltages. A single molecule entering the pore arouses transient fluctuation of the ion current, referred to as a current pulse, indicating a single molecule event of translocation. The monitored current pulses that are raised within the current baseline contribute to the physical features of the passing analytes and the dynamic interactions between the analytes and nanopores. The intensity, dwell time, capture frequency, and waveform of these current pulses can provide information on the dynamic changes in analytes including the volume, concentration, surface charge, and conformation features in solution [5,7,8,9]. Hence, it is important to collect and characterize the current pulses in nanopore measurements for a better understanding of the behavior of various analytes. However, the current pulse is instantaneous and is recorded by a high-gain and low-noise amplifier. The corresponding current pulses are weak and arise from the strong noise background of the ionic current trace. Thus, a well-defined current readout platform is a necessary component of the nanopore sensing in order to detect analytes and to characterize their properties, which will accelerate the nanopore development in modern diagnostic and bio-sensing fields. 

**Figure 1:**
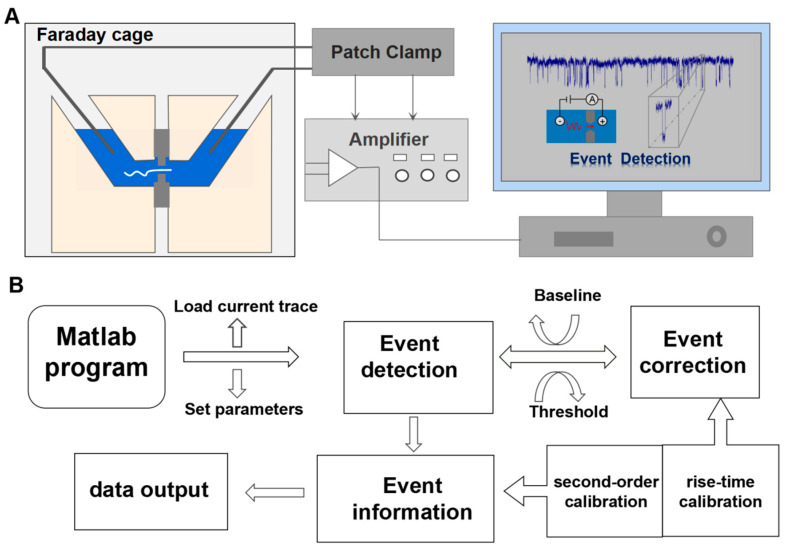
(**A**) The flow chart of nanopore current signals detection and processing by nanopore sensors; (**B**) the program design of the molecular pulse signal recognition of the nanopore sensor.

With the rapid development of nanopore sensing, there is great interest in developing tools and methods for robust data analysis within nanopore fields [5,10,11,12,13,14,15,16]. Currently, most of the data analyses have been performed with various open source and commercial software. In order to account for the complexity and diversity of the current signals, different techniques have been developed for the analysis of nanopore current traces. Firstly, denoising filters are often used in the software realization for already acquired digital data and signal amplification, including low-pass filters, Kalman filters, and wavelet transform [14,17,18,19,20]. To separate pulses from noise, Raillon’s group introduced OpenNanopore software, which is based on the cumulative sums algorithm to process the multi-level events in nanopore translocation [11]. Forstater’s group developed an improved data analysis tool called Modular Single-Molecule Analysis Interface (MOSAIC) for data measured from both biological and solid-state nanopores experiments based on two key algorithms: ADEPT for short-lived events and CUSUM^+^ for longer events [12]. Meanwhile, Sun’s group provided an automated adaptive and robust AutoNanopore platform for event detection in solid-state nanopore current traces with the highest coverage ratio [13,14]. Dekker et al. introduced a local baseline recalculation algorithm by iterative operation for separating DNA folded and unfolded states within translocation events [21]. Long et al. focused on an automatic and accurate nanopore data process with a second-order differential-based calibration method and an integration method to evaluate both the dwell time and current amplitude [22,23,24]. For baseline fitting profiles, Kim et al. proposed a clustering method (density-based spatial clustering of applications) to identify the boundaries of the event and for preliminary estimation of the levels within the events [25,26]. Recently, classical algorithms of machine learning have been proposed to improve the nanopore resolution [14,27,28,29,30]. Zhang et al. used deep learning based on a bi-path network (B-Net) for feature extraction of nanopore signals, which was capable of processing data with a low signal-to-noise ratio, far beyond that of the threshold-based algorithms [14,27]. Overall, these programs, which have been customized in a local laboratory, improve the quality of nanopore signal and accelerate the signal processing. However, they usually work well with specific targets in particular models, and the learning process, with great potential, usually runs in a large number of well-labeled data sets, which is challenging. Predictably, a more adaptable nanopore signal processing platform is required to explore the stochastic nature of a variety of molecule passage events in a complex environment. Here, an adaptive program of event detection and information extraction in nanopore measurement was designed based on the MATLAB programming interface. Figure 1A,B shows a flowchart of the nanopore current signals detection and the recognition program of the molecular pulse signals in the nanopore sensor. Clearly, the program is divided into several modules including molecular event detection, molecular event correction, event information, and data output. Firstly, the measured current trace can be directly loaded into the program by setting multi-parameters of local window width and double thresholds to identify the current pulse signals. Secondly, a set of the detected pulse signals is fitted and corrected to reduce the interference of background noise and the over coverage of pulse signals in the evaluation stage. Finally, the abundant information from the current signals is exported and saved for further quantitative analysis. These stages are interrelated and gradually progressive, with each step utilizing parameters from the previous step. The program is easily operated and broadly adapted to analyze large amounts of nanopore data with high quality and high throughput, which will accelerate nanopore sensing development in more specialized areas of rapid clinical diagnoses and optimal treatment regimens.

## 2. Materials and Methods

λ-DNA (TaKaRa, Co., Ltd. Dalian, China) was diluted in 1 M KCl at a pH of 8.0. All other chemical reagents used in the nanopore experiment were of analytical grade and use without further purification. The samples were prepared with Milli-Q super purified water with a resistance of >18 MΩ/cm. All solutions were filtered with a 0.02 μm Anotop filter (Whatman, Co. Maidstone, Kent, UK) before using. 

The DNA was detected by nanopore sensors. A patch clamp amplifier (Axon Instruments, Axopatch 700B) was used to measure the corresponding ionic current flowing through the nanopore as a function of the biased voltages. The sampling frequency was above 100 kHz with a low pass filter of 10 kHz cutoff frequency. The current signals were recorded by the 1440A digitizer (Molecular Devices, Inc. CA, USA). Data were collected over multiple experiments with the same nanopore. The whole nanopore device was set in a Faraday cage for shielding electromagnetic noise.

## 3. Results and Discussion

### 3.1. Nanopore Signal Processing Program

**Figure 2:**
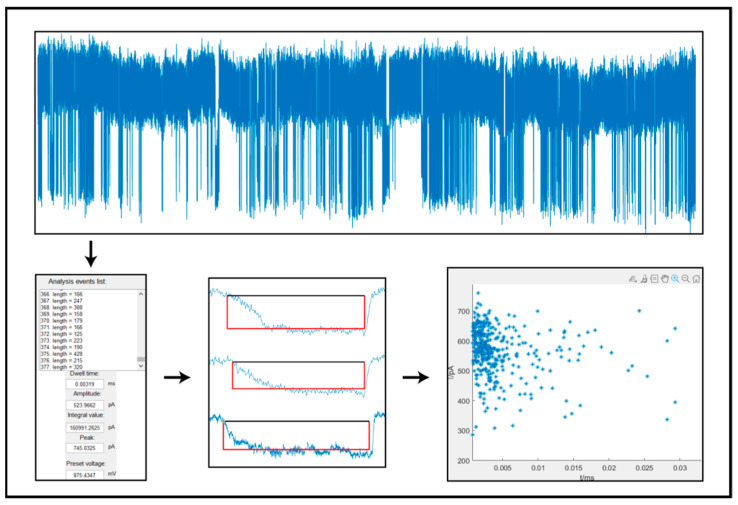
The nanopore signal processing interface on MATLAB program.

Because the high bandwidth recordings of nanopore sensors instantaneously produce a large amount of current signal data, it is necessary to process them in a programmed manner. Among digital signal software, MATLAB (MathWorks, Natick, MA, USA) is powerful software with the advantages of rapid operation on text, graphics, sound, and interactive features such as a human–machine interface. Thus, a signal processing program based on MATLAB was developed to extract molecular information from large amounts of nanopore data, as shown in Figure 2. Once the nanopore current signals were loaded into the program, a visual interaction interface of MATLAB was established and the selected current segment appeared in a movable data window. The preliminary detection was performed based on raw data by setting multiple parameters, including local window width and double thresholds. Here, the baseline current was considered a stable value in a short enough time, which is termed a local signal window. Therefore, the current signal trace recorded over a long time could be divided into a local window with a short enough time in order to track the baseline fluctuations, and the whole signal data fragment could be detected and analyzed by a moving the analysis window to minimize the effect of noise background, which improved the efficiency and accuracy of nanopore data analysis. Additionally, the determined current pulse signals were further corrected by a second-order differential function and rise time function in the main program. Next, the detected pulse signal was determined as a list of the molecular translocation events and individual signals could be checked in an enlarged graphics window. The feature parameters of these molecular events, including the dwell time, signal amplitude, peak value, and pulse signal integral area of the translocation events, were exported. Finally, the extracted information could be sorted into different populations with different criteria in the graph. The program’s self-adaptive and multiple standards were used to determine the current pulse signal of nanopore sensors in a moving data window, which significantly improved the statistical efficiency of nanopore data analysis. Meanwhile, more signal information, including peak values and current pulse areas, was helpful to better characterize the single molecular features of the analytes, such as shape, surface charges, volume, conformational change, and concentrations. 

### 3.2. Determination of Molecular Events

**Figure 3:**
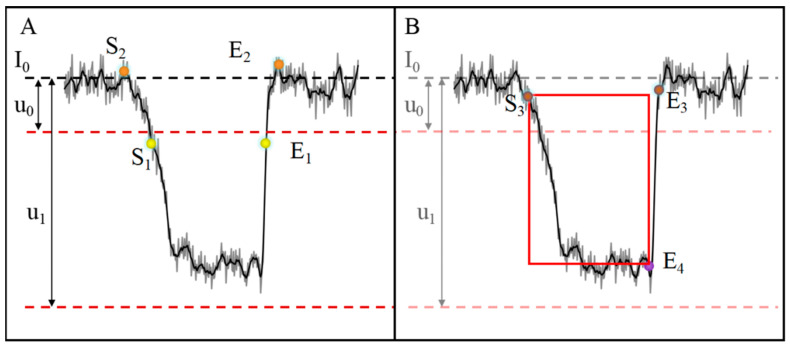
(**A**) The detection and (**B**) the correction of molecular events. S means the start point, and E means the end point.

As the biased voltage was added, a stable ionic current trace as recorded in the nanopore sensor. Once the target molecules were driven into the pore, the baseline current instantaneously fluctuated in the manner of current spike pulses. These transient spike pulses could be categorized as either a current reducing event with electrolyte conductance decreasing or current enhancing events with electrolyte conductance increasing generated from molecule translocations [5,8,10]. Because weak pulse signals in the picoampere and nanoampere range emerge in ionic current traces with a strong noise background, it is challenging to recognize the pulse signals. The typical pulse signals in gray raw line are shown in Figure 3A,B, and the current trace is fitted with a black line. When the current value of the detected data point in the window returned to the baseline current level, the translocation event came to an end. Meanwhile, the process involved multiple iterations and decoupling calculations of the local baseline based upon the threshold algorithm in order to remove the influence from the previous events. For the local stable baseline, the program ran throughout the whole current trace to evaluate the data points where the current was lower than the corresponding local threshold. The split strategy with a moving analysis window not only accelerated the signal processing efficiently but also improved the accuracy of the detected event information.

In an analysis window with a stable baseline current, the measured signal can be divided into three components using $I\left(t\right)=I_{0}\left(t\right)+\sum_{k=1}^{N}I_{\mathrm{event}-k}\left(t\right)+I_{n}\left(t\right)$, where $I_{0}\left(t\right)$ is the baseline current, $\sum_{k=1}^{N}I_{\mathrm{event}-k}\left(t\right)$ is the event current, and $I_{n}\left(t\right)$ is the current noise [14,25]. If the data point in the window is set to (i), the baseline current is the mean value of the current values of all data points in the window, set to baseline (i), the initial state of detection is i = 0, and the judgment starts from the first point after the window (i + 1). When the molecules enter the pores, the ionic current is transiently blocked. Therefore, the current value of the event on the translocation is less than the baseline current, and all testing data points of the event are between the double thresholds. As shown in Figure 3A, the baseline (I_0_) and double thresholds (the lower threshold u_0_ and the higher threshold u_1_) were defined to distinguish between signal and noise in a local window. Once the thresholds are set, the program searches the start point (S_1_) and end point (E_1_) in the entire traces. The start time (S_1_) is defined when a first level is observed away from the base current, and the event end time (E_1_) is defined when the signal crosses the base current value again. Considering the signal distortion due to noise filtering and digital-to-analog conversion, it is possible to miss some points in the initial position of the signal. In order to accurately cover all data points, the start point (S_2_) and the end point (E_2_) are reset by a tracking-back routine in MATLAB. Inevitably, the over-fitting of the pulse signal still causes a minor deviation in each event after back checking algorithm. Thus, the correction of the event is required to improve the measurement system.

### 3.3. Correction for Molecular Events

Typically, through-pore ionic current is collected with a range of different noise sources in a nanopore measurement. To better determine the molecular events, the current signal data were smoothed and corrected based on mathematical functions of the MATLAB program. First, the different selected fragments of the original current trace with low signal to noise ratio were fitted by Fourier functions, which are equivalent to the role of a low-pass filter. Smoothed signal data are preferable to identify the molecule events due to the reduction in background noise. After the smooth fitting of the electrical signals, the signal detection was analyzed using the correct functions including rise time correction and second-order difference correction. After the data fitting, the extreme points were searched by the correction function from the beginning to the end of the current pulses, as shown in Figure 3B. The extreme points of second-order difference correction were then recognized as the start point (S_3_) and the end point (E_3_). Due to the high band sampling and low-pass filter denoising, a rise time is required for a current pulse signal from the blocked state to the open state, and the pulse current is delayed back to baseline. In order to more precisely determine of the pulse signal model, the rise time correction function was used to reset to the local end point (E_4_), and the start location of the pulse event was the same (S_3_). Although the modified changes may appear minor, these corrected methods can improve the quality of the analysis result, especially for the pulses of fast translocation and bumping blockage.

### 3.4. Electrical Signal Feature Information Extraction

Once the current pulse signal of the nanopore sensor is determined, the method further extracts multiple parameters such as the residence time of the characteristic event, the signal pulse amplitude, and the signal integral area of the entire event; then, the data, are exported, which provides statistical and analytical maps of molecular event signature information. Generally, the basic theory of the nanopore-based detecting procedure mainly focuses on the amplitude (ΔI) and dwell time (Δt) of the current pulse signals. The signal amplitude of an abrupt current blockade on the baseline is associated with the volume and geometry of the molecule objects during translocation, and the dwell time is related to the dynamics of the objects within the pore. Analysis of these signatures can provide insight into the molecular structure, surface charge, and interaction between pore and molecules. Here, one more feature of peak areas was described by the time integral of the current amplitude from the baseline current, referred to as the event charge deficit (ECD), ∫ΔI(t)dt=ECD [31,32,33]. It is known that some nonspherical molecules passing through the pore in different conformations, such as DNA in a folded and unfolded state, induce compensating effects of decreased translocation time and increased current blockage for folded molecules. However, the integrated areas of all current pulses are equal for the same molecules in different conformations and same lengths. Thus, ECD is an important standard used to recognize individual molecules or particles in varied structures and orientations during translocation in a variable environment. The additional analysis is helpful for more accurately determining the molecular species and for analyzing the changes in molecular shape and structure in detail. Thus, the multi-parametric information extracted in our program provides a more comprehensive and accurate description of the analytes from nanopore current signals.

### 3.5. Applications to DNA Detection

**Figure 4:**
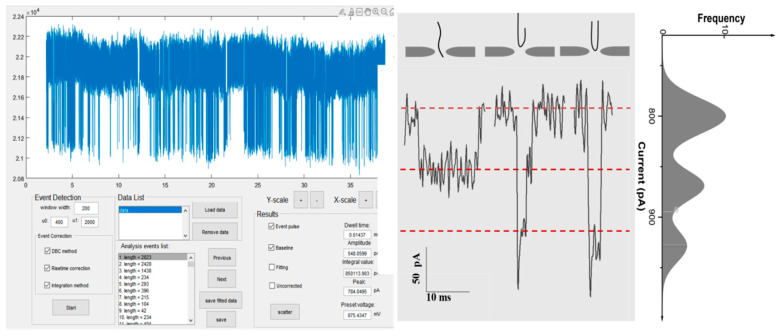
DNA detection by the nanopore sensor based on the MATLAB program.

Finally, the current signals of DNA translocation through nanopore were analyzed by the Event Detection program based on the MATLAB platform. A large amount of the transient current pulses generated by DNA passing through the pore was recorded in nanopore sensors, as shown in Figure 4. The features of the current amplitude, the dwell time, and the integral area of the pulses were analyzed by statistical function. As represented in Figure 4, three distinct dynamic translocation events were observed from the analysis of this method. The linear DNA molecules translocate over a longer period and a lower blockade current, while the folded ones more quickly pass through the nanopore and with a larger current blockage.

**Figure 5:**
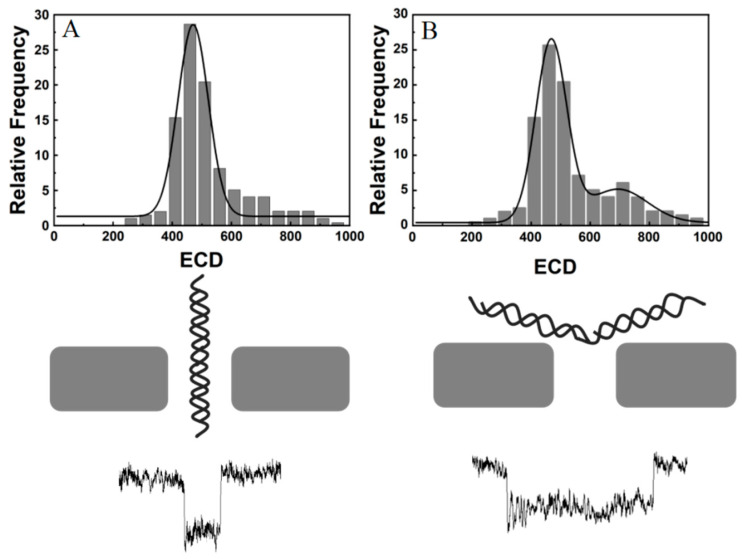
The ECD statistics of long DNA normal translocation through a nanopore (**A**) and plugging phenomenon mixed in translocation events in a nanopore (**B**).

In this demonstration of DNA translocation, the phenomenon of the agglomerations and packaging of long DNA trapped at the orifice of a nanopore is not obviously neglectable, which induces more pulse signals of longer duration and the amplitude changes expected from DNA. This phenomenon affects the capture statistics of pulse signals in nanopore sensing. In order to better distinguish the real signal of DNA translocation through a nanopore, an additional feature (event charge deficit (ECD)) was characterized with the amplitude (ΔI) and dwell time (Δt) of the current pulse signals in our study. It was confirmed that ECD, referred to as the integral area of obstructed ionic current over the duration of an event, is equal for the same class of DNA passing freely through the nanopore. For trapped molecules, the statistical analysis of ECD is different from that of DNA freely passing through the pore, as shown in Figure 5. As DNA molecules freely enter the pore in a linear and folded form, the ECD statistics follow a normal distribution model with one peak. More peaks appear in the ECD distribution in the presence of long DNA trapped in the pore. Therefore, our software, with comprehensive features of pulse signals, provides a better description of the dynamic process for DNA translocation, in coordination with the optimization experiment condition, to effectively reduce the DNA agglomeration phenomenon, including temperature, pH, voltages, etc. Therefore, the program will be useful for identifying and evaluating the diverse dynamic behavior of the objects interacting with nanopores at the single-molecule level.

## 4. Conclusions

Signal processing is an indispensable component of nanopore sensing. However, the current research focuses on nanopore equipment and multiple detection objects; the processing of the huge amount of data from complex nanopore signals is still not deep enough, which is a time-consuming and unstandardized process. Thus, a well-defined current readout platform is necessary to better detect analytes and characterize their properties. In our work, a robust data processing program of current pulse signals detected by nanopore sensors was developed based on the MATLAB platform. The program considers issues such as signal noise and baseline stability; the selected current segment was rapidly analyzed by the adaptive thresholds and corrected by multiple functions to reduce background noise in a moving local window, which greatly accelerated the processing efficiency of nanopore data analysis. Moreover, multi-dimensional information, such as the residence time of the detected pulse signal, the amplitude of the pulse signal, and the integral information of the pulse signal, was extracted and assessed, which will provide distinct scenarios of molecular translocation at the single-molecule level. Therefore, this automatic and accurate signal processing program can replace the artificial-assisted recognition, which is time-consuming and individually biased, and will promote the development of the nanopore sensing devices in various fields of diagnostic systems and precision medicine.

## Data Availability

Not applicable.
